# Joint application of hybrid iterative reconstruction and adaptive filters on neck‐and‐shoulder CT imaging: A clinical evaluation

**DOI:** 10.1002/acm2.13797

**Published:** 2022-10-14

**Authors:** Wenfeng Jin, Yifei Ma, Dan Han, Xiaojie Xie, Weiyuan Zhang, Yan Wu, Guozhi Zhang

**Affiliations:** ^1^ Department of Radiology First Affiliated Hospital of Kunming Medical University Kunming China; ^2^ United Imaging Healthcare Shanghai China

**Keywords:** adaptive filter, computed tomography, iterative reconstruction, streak artifacts

## Abstract

**Purpose:**

To assess whether the joint application of hybrid iterative reconstruction (HIR) and an adaptive filter (AF) could reduce streak artifacts and improve image quality of neck‐and‐shoulder computed tomography (CT).

**Methods:**

This study included 96 patients with suspicious neck lesions who underwent a routine nonenhanced scan on a 64‐slice CT scanner. The raw data were reconstructed using four different settings: filtered back projection (FBP), HIR, FBP + AF, and HIR + AF. Regions of interest were manually drawn in erector spine, axillary fat, latissimus dorsi, and dorsal cervical fat. Mean and standard deviation (SD) of the CT number, signal‐to‐noise ratio (SNR), and contrast‐to‐noise ratio (CNR) were obtained and compared using Wilcoxon signed‐rank tests. The qualitative assessments of five factors were compared by two independent investigators.

**Results:**

Compared to the other three settings, HIR + AF reduced noise in the area where the streak artifact of the lower neck were most serious (SD; all *p* ≤ 0.001). The SNR and CNR were improved significantly (all *p* ≤ 0.001). Compared to the other three settings, HIR + AF showed a significant improvement in CT image quality regarding the visibility of suspicious lesions, the extent of streaking artifacts, noise, soft‐tissue contrast, and visualization of small structures (all *p* ≤ 0.02).

**Conclusions:**

The combination of HIR and AF can significantly reduce streaking artifacts and improve image quality in neck‐and‐shoulder CT imaging.

## 1 INTRODUCTION

Thyroid cancer is the most prevalent endocrine malignancy.^1^ The incidence of thyroid cancer increased by 20% from 1990 to 2013.^2^ Computed tomography (CT) is widely used in the examination of thyroid cancer. Neck‐and‐shoulder CT images are often associated with streak artifacts. X‐rays must travel a relatively long distance and thus pass through strong attenuation within the shoulder in the lateral direction. As a result, few photons can reach the detector. When too few photons reach the detector, Poisson noise is amplified, causing streak artifacts in the images. This phenomenon is called photon starving. The artifacts may lower the local spatial resolution, mask lymph nodes, and obscure important diagnostic information, making thyroid cancer undistinguishable.^3^ Therefore, it is crucial to reduce or even eliminate artifacts in this region.

Several attempts have been made to suppress or remove the artifacts, including both algorithmic and nonalgorithmic methods. One nonalgorithmic method is tilting patients’ arms to prevent those bones with high attenuation from lining up in the transverse plane. However, it requires patient compliance, which might not always be easy in a practical setting.^4^, ^5^ Another nonalgorithmic method is to increase the dose via a higher tube voltage and/or higher tube current. Unfortunately, thyroid glands in the neck are sensitive to ionizing radiation. An increase in radiation exposure leads to an enhanced risk of the incidence of thyroid gland carcinoma, especially in children.^6^ As such, imposing a higher radiation dose to reduce streak artifacts for neck and shoulder, CT is not a practical option.^7^

**FIGURE 1:**
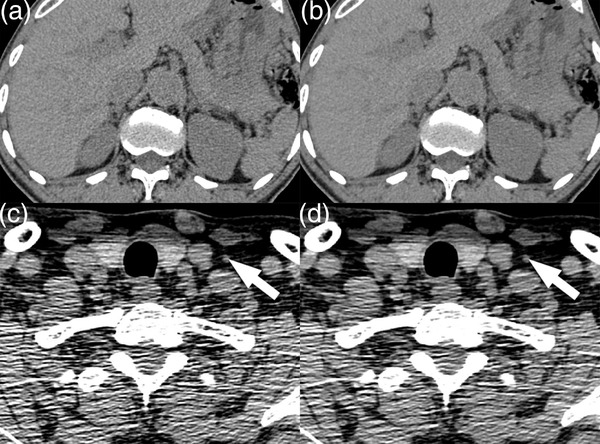
Abdominal images of a 69‐year‐old male reconstructed with filtered back projection (FBP) (a), hybrid iterative reconstruction (HIR) (b), shoulder images of a 46‐year‐old male with cervical lymph nodes (arrow) reconstructed with FBP (c), and HIR (d). Suppression of noise could be seen between (a) and (b), but the streak artifacts remained severe in (d)

Algorithmic methods, that is, advanced reconstruction algorithms and software‐based artifact correction algorithms for image reconstruction, have proven to be more effective than nonalgorithmic methods. They work on postprocessing the data and do not require any change in image acquisition. The first category of algorithmic methods is iterative reconstruction (IR). IR has developed rapidly in the past 10 years. Many studies have shown that IR can reduce noise and improve spatial resolution.^8^, ^9^ Several IR methods are available from major vendors, including model‐based IR (MBIR) and hybrid IR (HIR).^9^ MBIR requires additional computer resources and takes more time to reconstruct than filtered back projection (FBP). HIR is a combination of IR and FBP. It iteratively converts data from projection to image space by FBP and converts data from image to projection space by IR. It also iteratively filters data in both spaces separately.^9^ Moreover, the HIR used in this study implements system noise models into the IR technique and uses raw data in iterations to preserve the structure edges. As a result, it reduces the noise significantly, while preserving the edge information and maintaining the spatial resolution and structural information. HIR requires less data processing power than MBIR.^10^ Its speed is comparable to that of FBP. Although IR suppresses noise well, its ability to reduce streak artifacts remains unsatisfactory (Figure 1). The second category of algorithmic methods is deep learning image reconstruction (DLIR), which has emerged quite recently. DLIR learns from images without streak artifacts and reconstructs data with streak artifacts into images with low noise and few streak artifacts.^11^ However, as DLIR is only available on the latest premium‐end CT systems, not all imaging sites have access to this benefit.

The third category of algorithmic methods used is the artifact correction algorithm, data filter function in the raw data domain. The filter function recompenses photon noise at high absorption angles by customizing the filter bandwidth based on the ellipse shape of the cross section of the bone structure. The drawback of this algorithm is that successful modification of the filter depends on the patient's anatomy, which varies among individuals.^12^ Under‐ or overmodification may cause a reduction in spatial resolution.

An adaptive filter (AF) is a potentially efficient algorithm used to suppress or remove streak artifacts.^3^ AF technique is used to estimate the photon starvation phenomena based on the photon statistics. It is only applied to channels experiencing photon starvation. The strength of filtering is inversely proportional to the signal strength and is balanced between uniform noise texture and streaking artifact suppression. This kind of algorithm can filter differently among not only directions but also patients. It works well in both patients with and without streak artifacts. AF does not require much time and computer resources, and it can be flexibly combined with either FBP or HIR.^12^

In this study, the effect of the joint application of HIR and AF on neck‐and‐shoulder CT imaging was evaluated both quantitatively (in three regions of interest [ROIs]) and qualitatively (two independent raters) and compared with FBP + AF as well as FBP and HIR alone.

## 2 METHODS

The study was approved by the ethics committee of our institution. The requirement for informed consent was waived due to its retrospective nature.

### 2.1 Case selection

**FIGURE 2:**
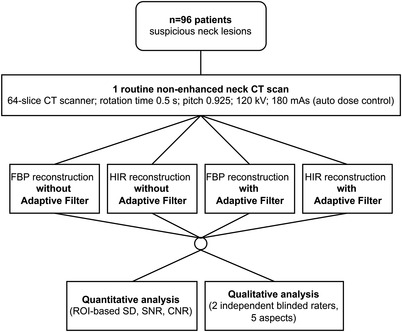
Flowchart of the study design

The sample size was calculated based on the results from a small‐scale preliminary experiment. Adult patients (>18 years) suspected of having neck lesions who had undergone neck‐and‐shoulder CT scans between January 2018 and September 2021 were selected for this study (Figure 2). A total of 96 patients (42 males and 54 females; mean age, 49.1 years; range, 19–87 years) were included in this study. This study was a single‐center study. Eligibility criteria were (a) patients who underwent neck‐and‐shoulder CT scans, (b) the presence of streak artifacts, and (c) available raw data for extra reconstruction. The exclusion criteria were (a) patients with metal implants and (b) the presence of motion artifacts.

### 2.2 Image acquisition

The patients were positioned with both arms down. All the images were acquired with a 64‐slice CT scanner (uCT 760, United‐Imaging Healthcare) using a standard protocol: 120 kV tube potential, 180 mA s reference tube current with dose modulation on (AutoDOM, United‐Imaging Healthcare), 0.925 spiral pitch, and 20 mm longitudinal collimation.

The median Dose Length Product of the scan was 453.3 mGy cm (range 200.9–1048.2 mGy cm), and the median CTDI_vol_ was 16.1 mGy (range 8.7–23.8 mGy).

### 2.3 Image reconstruction

The raw data were reconstructed using the following parameters: kernel, B SOFT B kernel; slice thickness, 2 mm; slice interval, 0.5 mm; and pixel matrix, 512 ⨯ 512. Each case was reconstructed using four different settings: FBP, HIR (KARL 3D, United‐Imaging Healthcare), FBP + AF (Adaptive Filter, United‐Imaging Healthcare), and HIR + AF. For HIR, the medium strength (strength, level 3) was applied. The AF implemented in the course of the reconstruction follows a similar theory to the one described in Hsieh et al.^12^ In FBP + AF, AF filtered the raw data in the projection domain, and then FBP generated reconstructed images. In HIR + AF, AF was involved in the iterations, which meant AF filtered the raw data in each iteration.

### 2.4 Quantitative analysis

One radiologist, with 10 years of diagnostic experience, was asked to draw three ROIs in areas with visually the maximum streak artifacts: ROI_1_, ROI_2_, and ROI_3_ were placed on the left erector spine, the axillary fat and the left latissimus dorsi, respectively. Another ROI_ref_ was placed on the dorsal cervical fat, which was not affected by the streak artifact and was therefore taken as a reference. All ROIs were ∼10 mm in diameter. The mean and standard deviation (SD) of the CT numbers were recorded. The signal‐to‐noise ratio (SNR) and contrast‐to‐noise ratio (CNR) were calculated as

(1) $$\mathrm{SNR}=\frac{\left|\mathrm{Mea}n_{\mathrm{ROI}}\right|}{SD_{\mathrm{ROI}}}$$

(2) $$\mathrm{CNR}=\frac{\left|\mathrm{Mea}n_{\mathrm{ROI}}-\mathrm{Mea}n_{\mathrm{reference}}\right|}{\left(SD_{\mathrm{ROI}}+SD_{\mathrm{reference}}\right)/2}$$

### 2.5 Qualitative analysis

Two radiologists, one with 10 years and the other with 13 years of diagnostic experience, were blinded to the reconstruction methods and all the information about each patient (i.e., pathology and clinical history) when performing the qualitative analysis. The analysis was conducted on the clinical workstation (UIdeal, United‐Imaging Healthcare), in exactly the same reading environment as in the clinical workflow, where the images were first presented in the soft‐tissue window (W/L = 300/30 HU) and the raters were allowed to adjust all common display parameters. The following qualitative assessments were performed: the visibility of the suspicious lesions (1 = unacceptable, …, 5 = excellent), noise level (1 = unacceptable, …, 5 = minimal), soft‐tissue contrast (1 = very poor, …, 5 = excellent), visualization of small structures (1 = unacceptable, …, 5 = excellent), and streak artifacts (1 = major artifacts, …, 5 = no artifacts).^13^ The final scores were averaged between the two radiologists.

### 2.6 Statistical analysis

The normality evaluation of the quantitative measurements in each group was performed by using the Shapiro–Wilk test. Then, a paired *t* test was performed on the normally distributed quantitative measurements. The Wilcoxon signed‐rank test was performed on the ordinal qualitative measurements and nonnormally distributed quantitative measurements between the HIR + AF group and the HIR group, the HIR + AF group and the FBP + AF group, and the HIR + AF group and the FBP group. The consistency between the two raters was evaluated using Cohen's kappa statistics. The scale of Cohen's kappa statistics was as follows: less than 0.20, poor; 0.21–0.40, fair; 0.41–0.60, moderate; 0.61–0.80, good; and 0.81–1.00, excellent agreement. *p* ≤ 0.05 was considered statistically significant. All statistical analyses were performed using Python version 3.8.8 (python.org) with SciPy package version 1.7.1 (SciPy.org) and scikit‐learn package version 0.24.2 (scikit‐learn.org).

## 3 RESULTS

### 3.1 Qualitative image quality

**FIGURE 3:**
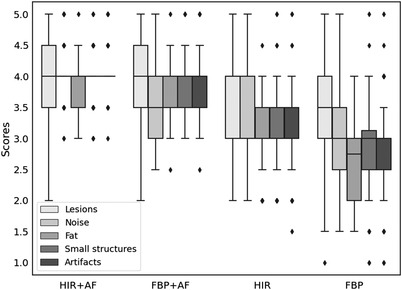
Subjective scores of visibility of suspicious lesions, the noise of the image, soft‐tissue contrast, visualization of small structures, and the extent of streaking artifacts on a five‐point Likert scale (1, poor; 5, excellent)

**FIGURE 4:**
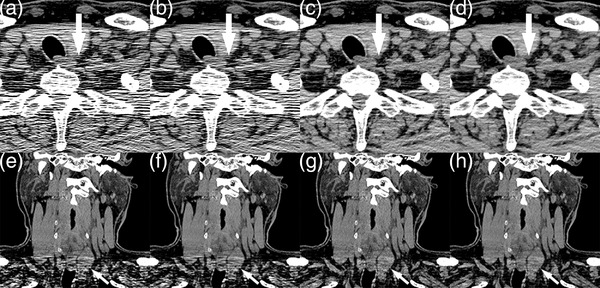
Images of a 67‐year‐old male with a thyroid nodule (arrows) in the left thyroid lobes from the filtered back projection (FBP) (a and e), hybrid iterative reconstruction (HIR) (b and f), FBP + adaptive filter (AF) (c and g), and HIR + AF (d and h) groups

**FIGURE 5:**
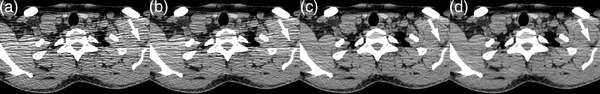
Images of a 27‐year‐old male with a space‐occupying lesion (white arrows) at the root of the left neck from the filtered back projection (FBP) (a), hybrid iterative reconstruction (HIR) (b), FBP + adaptive filter (AF) (c), and HIR + AF (d) groups

Qualitative scores are presented in Figure 3. Examples are presented in Figures 4 and 5. We used box plots to present the distributions of the scores because the scores did not follow normal distributions. Among the four groups, the HIR + AF group exhibited the highest qualitative scores for all five assessments (all median scores were 4, *p* ≤ 0.001) with the narrowest interquartile range. The scores for noise level, visualization of small structures, and streak artifacts in the HIR + AF group had such narrow distributions around the median score of 4 that the 25% quantile, median, and 75% quantile overlapped with each other. The result of the Cohen's kappa test was *κ* = 0.47, showing fair agreement.

### 3.2 Quantitative image quality

There was at least one group of measurements that did not follow the normal distribution in all four measurements (CT, SD, SNR, CNR), so we performed the Wilcoxon signed‐rank test on all measurements. The results of the quantitative analysis (Table 1) showed that mean CT numbers of all four ROIs were significantly different among four groups (all *p* ≤ 0.03) because varying degrees of streak artifacts notably changed the local CT numbers. SD of CT numbers of all four ROIs in the HIR + AF group was the lowest among all four groups (all *p* ≤ 0.001). SNR and CNR of ROI_1–3_ in the HIR + AF group were the highest among all four groups (all *p* ≤ 0.001). Compared to the FBP + AF, HIR, and FBP groups, the HIR + AF group showed a 21.4%, 21.8%, and 51.1% increased median SNR as well as a 19.0%, 17.7%, and 38.2% increased median CNR at the erector spine, respectively. At axillary fat, the increases were 17.8%, 38.4%, and 68.9% for the median SNR and 18.1%, 29.7%, and 55.6% for the median CNR, respectively. At the latissimus dorsi, the increases were 29.5%, 19.9%, and 55.5% for the median SNR and 20.6%, 11.9%, and 34.4% for the median CNR, respectively. Compared between ROIs, the SNR and CNR between the HIR + AF group and the FBP group showed the largest increase at axillary fat.

**TABLE 1:** Quantitative measurements for the filtered back projection (FBP), hybrid iterative reconstruction (HIR), FBP + adaptive filter (AF), and HIR + AF image groups

	FBP	HIR	FBP + AF	HIR + AF
Erector spinae				
Mean (HU)	49.9	49.7	48.8	48.7
SD (HU)	24.5	19.7	17.8	14.5
SNR	2.3	2.9	2.9	3.6
CNR	6.0	7.3	7.1	8.6
Axillary fat				
Mean (HU)	–94.1	–93.9	–96.1	–96.0
SD (HU)	27.5	22.4	18.8	15.7
SNR	4.1	5.0	5.5	6.7
CNR	1.1	1.2	1.4	1.5
Latissimus dorsi				
Mean (HU)	54.5	54.4	54.3	54.2
SD (HU)	17.9	14.1	14.4	11.3
SNR	3.4	4.3	4.0	5.1
CNR	7.3	8.8	8.1	10
Dorsal cervical fat				
Mean (HU)	–79.3	–80.9	–79.6	–81.2
SD (HU)	21.6	18.4	21.3	16.7

Abbreviations: CNR, contrast‐to‐noise ratio; SD, standard deviation; SNR, signal‐to‐noise ratio.

## 4 DISCUSSION

This study aimed to assess whether using HIR with a commercially available AF could improve image quality in terms of streak artifact reduction and noise suppression for unenhanced neck and shoulder CT imaging. Our results indicated that compared to using HIR only or FBP with an AF, using HIR with an AF significantly improved the image quality. The improvement in the SNR and CNR was consistent with studies on HIR^14^ and AF.^12^

However, the presence of bright streak artifacts may cause a higher SNR and CNR by elevating local CT numbers. Therefore, compared to the other three groups, although streak artifacts were suppressed in the HIR + AF group, the increases in SNR and CNR might partly result from the different distributions of streak artifacts^15^ due to varying algorithm logic between FBP and HIR.

Consistent with the objective evaluations, the subjective evaluations of both raters supported that compared to the other methods, the HIR with AF method improved the image quality according to assessments of the visibility of suspicious lesions, noise level, soft‐tissue contrast, visualization of small structure, and streak artifacts (*p* ≤ 0.01). Previous studies also confirmed this trend.^16^, ^17^

Neck‐and‐shoulder CT data of two pediatric (6 and 12 years) and two adolescent (14 and 17 years) patients were found in the study period during data collection. As we decided to focus on adult patients, they were not included in the first place. The evaluation of these four cases revealed the same tendency as the adult: The HIR + AF image obtained the highest subjective scores, SNR, and CNR as well as the lowest noise. However, we cannot come to a statistically significant conclusion for pediatric or adolescent patients due to the limited number of cases.

Many previous studies of the reduction of streak artifacts have focused on metal implants^15^, ^18^ and deep learning.^19^ However, our study focused on reducing the streak artifacts caused by bones and the method that can be used in an economic CT system.

AF does not require heavy computing power; therefore, it has the flexibility to be made available on economic CT systems, including 16‐ and 4‐slice CT. HIR with AF does not require an extra dose or manual operation. The typical speed for HIR + AF reconstruction of neck‐and‐shoulder CT is within 1 min. Consequently, it was easily added to the daily clinical workflow.

Previous research shows that MBIR and DLIR suppress artifacts when compared to FBP.^20^, ^21^ Because AF works in the raw data domain, it may have the flexibility to work together with MBIR or DLIR. However, in this study, we did not compare the performance of MBIR or DLIR with AF. Further study on this point could also be of interest. The performance of HIR with AF in other parts of the body is still not clear. The evaluation of the image quality in pelvic CT images, which is also highly associated with streak artifacts, might be the next area of investigation.

In conclusion, the application of AF in the course of image reconstruction can significantly reduce streak artifacts and improve the overall image quality in neck and shoulder CT imaging. The combined use of HIR and AF performs better than FBP with AF or HIR alone.

## CONFLICT OF INTEREST

No conflicts of interest.

## AUTHOR CONTRIBUTION

Wenfeng Jin, Yifei Ma, Xiaojie Xie, Weiyuan Zhang, and Yan Wu contribute to the acquisition, analysis, and interpretation of data and drafting the work. Dan Han and Guozhi Zhang contribute to revising the work critically for important intellectual content. All authors contribute to the conception and design of the work. They approve the final version to be published and agree to be accountable for all aspects of the work.
